# The role of PAQR3 in cancer progression—Molecular regulation, signaling pathways, and clinical implications: A review

**DOI:** 10.17305/bb.2026.13696

**Published:** 2026-01-29

**Authors:** Yan Lv, Dan Li, Xiao-Fei Ren, Qiang Guo, Qiao-Ya Ren

**Affiliations:** 1 Department of Cardiothoracic Surgery, The Second Affiliated Hospital of Zunyi Medical University, Zunyi, China; 2 Department of Oncology, Taihe Hospital, Hubei University of Medicine, Shiyan, China; 3 Department of Cardiothoracic Surgery, Taihe Hospital, Hubei University of Medicine, Shiyan, China; 4 Department of Oncology, The Second Affiliated Hospital of Zunyi Medical University, Zunyi, China

**Keywords:** *PAQR3*, cancer, miRNA, prognosis, 5-Aza-CdR

## Abstract

Progesterone and adiponectin receptor 3 (PAQR3) is a Golgi-localized seven-transmembrane protein that anchors rapidly accelerated fibrosarcoma kinase (Raf) and suppresses rat sarcoma/rapidly accelerated fibrosarcoma/mitogen-activated protein kinase kinase/extracellular signal-regulated kinase (Ras/Raf/MEK/ERK) signaling, thereby influencing cellular proliferation, differentiation, and metastasis. This review aims to summarize the expression patterns, regulatory mechanisms, key downstream pathways, and clinical significance of *PAQR3* in cancer. We synthesized findings from published clinical and experimental studies, including *in vitro* assays and nude mouse xenograft models, that evaluate *PAQR3* expression, function, and signaling interactions across various tumor types. Overall, *PAQR3* is frequently downregulated in many cancers, potentially due to promoter methylation, and low expression levels are associated with adverse clinicopathologic features and reduced survival. Functionally, *PAQR3* overexpression inhibits proliferation, colony formation, migration, invasion, and tumor growth, primarily through the inhibition of extracellular signal-regulated kinase (ERK) and phosphatidylinositol 3-kinase/protein kinase B (PI3K/AKT) pathways and modulation of epithelial-mesenchymal transition (EMT). Additionally, PAQR3 is linked to nuclear factor kappa B/tumor protein p53 (NF-κB/p53), epidermal growth factor/beta-catenin (EGF/β-catenin) signaling, autophagy, and nuclear factor erythroid 2–related factor 2/ferroptosis (Nrf2/ferroptosis). These effects are modulated by upstream regulators, including microRNA-543 (miR-543), circular RNA 0043280/microRNA-203a-3p (circ_0043280/miR-203a-3p), microRNA-15b (miR-15b), human epidermal growth factor receptor 2 (HER2), 5-aza-2′-deoxycytidine (5-Aza-CdR), autophagy-related 7 (ATG7), and damage-specific DNA binding protein 2 (DDB2). In conclusion, PAQR3 functions as a tumor suppressor and holds potential as a prognostic biomarker. Targeting PAQR3-related pathways may provide new therapeutic opportunities.

## Introduction

Numerous studies have demonstrated that abnormal gene expression is associated with cancer cell growth, metastasis, and poor prognosis in cancer patients [1–6]. Progestin and AdipoQ Receptor 3 (*PAQR3*) is a gene located on human chromosome 4 that encodes a membrane protein characterized by a unique seven-transmembrane domain architecture. This protein is predominantly localized in the Golgi apparatus and plays a critical role in intracellular signal transduction. It functions by anchoring Raf kinase to the Golgi membrane, thereby exerting a negative regulatory effect on the Ras/Raf/MEK/ERK signaling cascade. *PAQR3* influences essential cellular processes, including proliferation and differentiation, highlighting its significance as a key regulator in maintaining normal cellular physiology through Rat sarcoma (Ras)/Rapidly Accelerated Fibrosarcoma (Raf)/mitogen-activated protein kinase kinase (MEK)/extracellular signal-regulated kinase (ERK) signaling [6]. Recent research has confirmed that the downregulation of *PAQR3* is linked to various aspects of cancer biology, such as cell proliferation, migration, and invasion across multiple cancer types [7–36]. Despite significant advancements in cancer treatment, the complexity of cancer cell biology, particularly the role of specific genes like *PAQR3*, continues to present challenges in developing more effective and targeted therapies. This review provides an update and expansion relative to existing synthesizations, including recent findings from 2023–2025 that were not addressed by Guo et al., and encompasses a broader range of literature than the meta-analysis conducted by Zhai et al. [34, 35]. The aim of this review is to summarize the roles, mechanisms, and clinical significance of *PAQR3* in cancer, offering new insights and potential therapeutic targets for clinical practitioners and researchers.

### Expression levels of PAQR3 significantly decrease in cancer

The expression levels of *PAQR3* are significantly reduced in various cancer tissues and cell lines [7–27]. Specifically, compared to normal tissues, *PAQR3* expression is notably lower in tissues from osteosarcoma, thyroid cancer, glioma, lung cancer, hepatocellular carcinoma, cervical cancer, colorectal cancer, diffuse large B-cell lymphoma, acute lymphoblastic leukemia, breast cancer, esophageal cancer, gastric cancer, and renal cell carcinoma (Table 1). This decline in expression is also observed in osteosarcoma, glioma, lung cancer, hepatocellular carcinoma, diffuse large B-cell lymphoma, breast cancer, esophageal cancer, and gastric cancer cells (Table 1). The downregulation of *PAQR3* in cancer cells may be attributed to epigenetic modifications. Specifically, DNA methylation of the *PAQR3* promoter region has been implicated in certain cancer types, leading to reduced gene transcription and subsequently lower protein expression levels [10, 22]. Preliminary evidence suggests that *PAQR3* may act as a tumor suppressor gene in cancer progression.

**Table 1:** Expression of PAQR3 in cancer

**Type**	**N**	**Tissues**	**Cells**	**Cancer cells**	**Normal cell**	**Ref**
Osteosarcoma	60	Down	Down	SOSP-9607, SAOS-2, MG63, U2OS	hFOB	[7]
Thyroid cancer	60	Down	-	-	-	[8]
Glioma	11	Down	Down	U251, U87, LN-18	1800	[9]
Lung cancer	106	Down	-	-	-	[10]
Lung cancer	60	Down	-	-	-	[11]
Lung cancer	20	Down	Down	SK-MES-1, A549, SPCA-1, H1229	BEAS-2B	[12]
Hepatocellular carcinoma	60	Down	Down	HepG2, Hep3B, Bel7402, SMMC-7721	HL-7792	[13]
Hepatocellular carcinoma	194	Down	-	-	-	[14]
Cervical cancer	40	Down	-	-	-	[15]
CRC	54	Down	-	-	-	[16]
CRC	62	Down	-	-	-	[17]
DLBCL	46	Down	Down	SUDHL4, OCI-LY19, U2932, OCI-LY10	HMy2.CIR	[18]
ALL	43	Down	-	-	-	[19]
Breast cancer	60	Down	Down	MDA-MB-231, BT474, SKBR3, MCF7	MCF-10A	[20]
Breast cancer	82	Down	-	-	-	[21]
Breast cancer	46	Down	-	-	-	[22]
ESCA	40	Down	Down	KYSE150, ECA-109, TE-1	HEsEpiCs	[23]
ESCA	80	Down	-	-	-	[24]
ESCA	-	-	Down	EC9706, TE13, ECA109	HEEC	[25]
Gastric cancer	166	Down	Down	HGC27, SGC7901	GES-1	[26]
Gastric cancer	300	Down	-	-	-	[27]
RCC	31	Down	-	-	-	[28]

Abbreviations: CRC: Colorectal cancer; RCC: Renal cell carcinoma; ESCA: Esophageal cancer; ALL: Acute lymphoblastic leukemia; DLBCL: Diffuse large B-cell lymphoma.

### Overexpression of PAQR3 inhibits cancer cell growth and metastasis

Research has confirmed that inhibiting oncogene expression or promoting tumor suppressor gene expression can suppress cancer growth and metastasis [34–36]. *PAQR3* is frequently downregulated in cancer tissues and cells, and its activation can inhibit cancer cell growth (Table 2). Specifically, *PAQR3* can suppress the proliferation of osteosarcoma, glioma, lung cancer, hepatocellular carcinoma, cervical cancer, colorectal cancer, diffuse large B-cell lymphoma, acute lymphoblastic leukemia, breast cancer, esophageal cancer, gastric cancer, renal cell carcinoma, colon cancer, and prostate cancer cells [7, 9, 11–15, 17–19, 21, 23, 25–32, 36]. Moreover, *PAQR3* overexpression suppresses colony formation in lung cancer, hepatocellular carcinoma, colorectal cancer, breast cancer, esophageal cancer, gastric cancer, colon cancer, and prostate cancer cells [11, 14, 17, 21–23, 26, 27, 29, 30, 32, 36]. Additionally, *PAQR3* overexpression can inhibit cell cycle progression in lung cancer and esophageal cancer cells [11, 12, 23]. Furthermore, *PAQR3* promotes apoptosis in lung cancer and acute lymphoblastic leukemia cells [11, 12, 19].

**Table 2:** The role of PAQR3 in cancer cell growth *in vitro*

**Type**	**Cancer cells**	**Proliferation**	**Clone formation**	**Cycle**	**Apoptosis**	**Ref**
Osteosarcoma	MG63	Inhibition	-	-	-	[7]
Glioma	U251, U87	Inhibition	-	-	-	[9]
Lung cancer	A549, H1299	Inhibition	Inhibition	Inhibition	Promotion	[11]
Lung cancer	A549, H1299	Inhibition	-	Inhibition	Promotion	[12]
Hepatocellular carcinoma	HepG2	Inhibition	-	-	-	[13]
Hepatocellular carcinoma	Hep-3B	Inhibition	Inhibition	-	-	[14]
Cervical cancer	MS751, HeLa	Inhibition	-	-	-	[15]
Colorectal cancer	SW-480	Inhibition	Inhibition	-	-	[17]
DLBCL	SUDHL4, U2932	Inhibition	-	-	-	[18]
ALL	CEM-C1, Jurkat	Inhibition	-	-	Promotion	[19]
Breast cancer	MCF7, SKBR3, MDA-MB-231, MDA-MB-468, MDA-MB-453	Inhibition	Inhibition	-	-	[21]
Breast cancer	MCF-7	-	Inhibition			[22]
ESCA	ECA-109, TE-1	Inhibition	Inhibition	Inhibition	-	[23]
ESCA	ECA-109	Inhibition				[25]
Gastric cancer	HGC27	Inhibition	Inhibition	-	-	[26]
Gastric cancer	AGS	Inhibition	Inhibition	-	-	[27]
RCC	HUVEC	Inhibition	-	-	-	[28]
Lung cancer	HCC827	Inhibition	Inhibition	-	-	[29]
Prostate cancer	PC3, DU145	Inhibition	Inhibition	-	-	[30]
Gastric cancer	BGC-823, SGC-7901	Inhibition	-	-	-	[31]
Gastric cancer	AGS	Inhibition	Inhibition	-	-	[32]
Colon Cancer	HCT116, HCT115	Inhibition	Inhibition	-	-	[36]

Abbreviations: CRC: Colorectal cancer; RCC: Renal cell carcinoma; ESCA: Esophageal cancer; ALL: Acute lymphoblastic leukemia; DLBCL: Diffuse large B-cell lymphoma.

Activation of *PAQR3* expression can also suppress cancer cell metastasis (Table 3). Specifically, results from Transwell and wound healing assays indicate that *PAQR3* overexpression can inhibit the migration of osteosarcoma, glioma, cervical cancer, breast cancer, esophageal cancer, gastric cancer, renal cell carcinoma, colon cancer, and prostate cancer cells [7, 9, 15, 20–23, 25–33, 36]. Additionally, *PAQR3* overexpression inhibits the invasion of osteosarcoma, glioma, hepatocellular carcinoma, cervical cancer, breast cancer, esophageal cancer, and gastric cancer cells [7, 9, 13, 15, 22, 23, 25, 31]. Notably, *PAQR3* overexpression inhibits the tube formation ability in renal cell carcinoma cells [28]. Based on this information, it can be preliminarily concluded that *PAQR3* functions as a tumor suppressor *in vitro*, and enhancing *PAQR3* expression may delay cancer cell growth and metastasis.

**Table 3:** The role of PAQR3 in cancer cell metastasis *in vitro*

**Type**	**Cancer cells**	**Migration**	**Invasion**	**Wound healing**	**Tube formation**	**Ref**
Osteosarcoma	MG63	Inhibition	Inhibition	-	-	[7]
Glioma	U251, U87	Inhibition	Inhibition	-	-	[9]
Hepatocellular carcinoma	HepG2	-	Inhibition	-	-	[13]
Cervical cancer	MS751, HeLa	Inhibition	Inhibition	Inhibition	-	[15]
Breast cancer	MDA-MB-231	Inhibition	Inhibition	-	-	[20]
Breast cancer	MCF7, SKBR3, MDA-MB-231, MDA-MB-468, MDA-MB-453	Inhibition	-	Inhibition	-	[21]
Breast cancer	MCF-7	-	Inhibition	-	-	[22]
ESCA	ECA-109, TE-1	-	Inhibition	-	-	[23]
ESCA	ECA-109	Inhibition	Inhibition	-	-	[25]
Gastric cancer	HGC27	Inhibition		Inhibition	-	[26]
Gastric cancer	AGS	Inhibition	-	Inhibition	-	[27]
RCC	HUVEC	Inhibition	-	Inhibition	Inhibition	[28]
Prostate cancer	PC3, DU145	Inhibition	-	Inhibition	-	[30]
Gastric cancer	BGC-823, SGC-7901	-	Inhibition	Inhibition	-	[31]
Gastric cancer	AGS	Inhibition	-	Inhibition	-	[32]
Gastric cancer	AGS	Inhibition	-	-	-	[33]
Colon Cancer	HCT116, HCT115	Inhibition	-	-	-	[36]

Abbreviations: RCC: Renal cell carcinoma; ESCA: Esophageal cancer.

Activation of *PAQR3* expression in glioma, cervical cancer, colorectal cancer, diffuse large B-cell lymphoma, esophageal cancer, lung cancer, and gastric cancer cells, followed by the establishment of tumorigenic models in athymic nude mice, demonstrated that *PAQR3* overexpression significantly inhibits tumor growth and proliferation in these models [9, 15, 17, 18, 23, 25, 29, 33]. This finding suggests that *PAQR3* functions as a tumor suppressor *in vivo* (Table 4), and enhancing *PAQR3* expression could represent a viable therapeutic strategy in clinical settings. However, further research is necessary to develop safe and effective methods to specifically upregulate *PAQR3* expression in human cancer patients without causing significant side effects.

**Table 4:** The role of PAQR3 in the tumorigenic potential of cancer cells in nude mice *in vivo*

**Type**	**Cancer cells**	**Roles**	**Ref**
Glioma	U251	Inhibition	[9]
Cervical cancer	-	Inhibition	[15]
CRC	SW-480	Inhibition	[17]
DLBCL	U2932	Inhibition	[18]
ESCA	ECA109	Inhibition	[23]
ESCA	ECA109	Inhibition	[25]
Lung cancer	HCC827	Inhibition	[29]
Gastric cancer	AGS	Inhibition	[33]

Abbreviations: CRC: Colorectal cancer; ESCA: Esophageal cancer; DLBCL: Diffuse large B-cell lymphoma.

## PAQR3 involves the signaling mechanisms

### Upstream regulation of PAQR3 in cancer

*PAQR3* may be inhibited by upstream circular RNA/microRNAs (miRNAs), thereby affecting the function of the target genes of *PAQR3* [13, 15, 20, 31]. For instance, Yu et al. reported that miR-543 is overexpressed in hepatocellular carcinoma tissues, while *PAQR3* is downregulated, establishing a negative correlation and a targeted regulatory relationship between the two. miR-543 can promote the proliferation and invasion of hepatocellular carcinoma cells by specifically targeting and suppressing *PAQR3* expression [13]. Zhang et al. noted a significant decrease in circ_0043280 levels in cervical cancer; circ_0043280 can competitively bind to miR-203a-3p, thereby promoting *PAQR3* expression and suppressing the growth and metastasis of cervical cancer [15]. Qi et al. demonstrated a targeted regulatory relationship between miR-15b and *PAQR3*, where miR-15b inhibits *PAQR3* expression, promoting progression in breast and gastric cancers [20, 31]. Additionally, *PAQR3* may be regulated by factors such as Human epidermal growth factor receptor 2 (HER2), 5-Aza-2′-deoxycytidine (5-Aza-CdR), Autophagy related 7 (ATG7), P6-55, and Damage-specific DNA binding protein 2 (DDB2) [19, 21–23, 29, 32, 36]. For example, Li et al. reported that *PAQR3* can inhibit the proliferation, colony formation, and migration of breast cancer cells. Trastuzumab, which inhibits HER2 expression, can promote *PAQR3* expression, thereby enhancing its inhibitory effects on breast cancer [21]. Chen et al. demonstrated that treatment with 5-Aza-CdR significantly stimulates *PAQR3* expression in breast and esophageal cancer cells, thereby enhancing *PAQR3*’s efficacy against cancer cells [22, 23].

## Downstream effects of PAQR3 in cancer

### The PAQR3/Ras/Raf/MEK/ERK signaling axis

**Figure 1.**
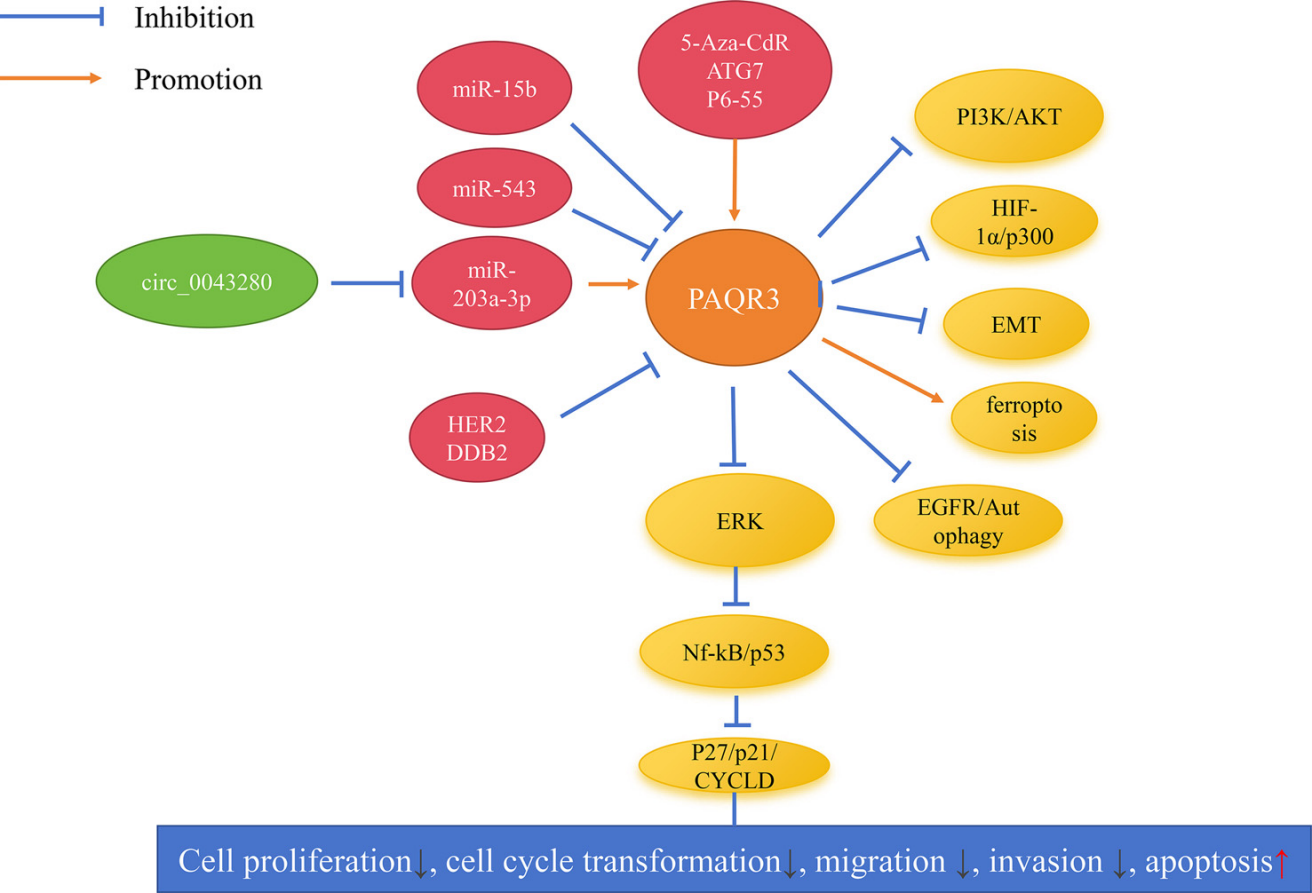
**PAQR3-centered signaling network.** Schematic summary of reported upstream regulators and downstream effectors of PAQR3 in cancer, with emphasis on the PAQR3/Ras/Raf/MEK/ERK axis discussed in the text. Upstream non-coding RNAs (circ_0043280; miR-15b, miR-543, miR-203a-3p) and protein/epigenetic regulators (HER2/ERBB2, DDB2; 5-Aza-2′-deoxycytidine (5-Aza-CdR), ATG7, p6-55) modulate PAQR3 expression/activity. Functionally, PAQR3 attenuates ERK signaling (including reduced ERK phosphorylation reported in multiple tumor models) and influences downstream modules such as NF-κB/p53 and cell-cycle regulators (p21/CDKN1A, p27/CDKN1B, cyclin D1), thereby restraining tumor cell proliferation, cell-cycle progression, migration and invasion, while promoting apoptosis. PAQR3 also intersects with additional pathways implicated in tumor biology, including PI3K/AKT, HIF-1α/p300, EMT, EGFR-linked autophagy, and ferroptosis (context-dependent). Blue blunt-ended lines indicate inhibition; orange arrows indicate activation/promotion. Abbreviations: PAQR3: Progestin and adipoQ receptor family member 3; circ_0043280: Circular RNA circ_0043280; miR: MicroRNA; HER2/ERBB2: Human epidermal growth factor receptor 2; DDB2: Damage-specific DNA binding protein 2; 5-Aza-CdR: 5-Aza-2′-deoxycytidine; ATG7: Autophagy related 7; EGFR: Epidermal growth factor receptor; ERK: Extracellular signal-regulated kinase; NF-κB: Nuclear factor kappa B; p53: Tumor protein p53; CDKN1A/p21: Cyclin-dependent kinase inhibitor 1A; CDKN1B/p27: Cyclin-dependent kinase inhibitor 1B; PI3K: Phosphatidylinositol 3-kinase; AKT: AKT serine/threonine kinase; HIF-1α: Hypoxia-inducible factor-1 alpha; EP300/p300: Histone acetyltransferase p300; EMT: Epithelial–mesenchymal transition.

ERK, a member of the Mitogen-Activated Protein Kinase (MAPK) family, plays a pivotal role in the signaling network governing cell growth, development, and division [37]. *PAQR3* has been shown to inhibit the growth and metastasis of various cancers, including osteosarcoma, cervical cancer, esophageal cancer, gastric cancer, renal cell carcinoma, colorectal cancer, and prostate cancer, by suppressing ERK signaling [7, 15, 17, 23, 25, 27, 28, 30, 32], as illustrated in Table 5 and Figure 1. For example, Ma et al. demonstrated that overexpression of *PAQR3* suppresses the phosphorylation of ERK (p-ERK), thereby inhibiting the proliferation and invasion of osteosarcoma cells. Notably, the MEK inhibitor U0126 completely negates the effects of *PAQR3* silencing on osteosarcoma cell proliferation and invasion [7]. Bai et al. reported that *PAQR3* overexpression reduces p-ERK protein levels in esophageal cancer cells and enhances the expression of downstream ERK proteins, such as Cyclin-dependent kinase inhibitor 1B (CDKN1B) and Cyclin-dependent kinase inhibitor 1A (CDKN1A), while inhibiting cyclin D1, Cyclin-dependent kinase 4 (CDK4), and Cyclin-dependent kinase 2 (CDK2), thus suppressing esophageal cancer growth [23, 25]. Additionally, *PAQR3* overexpression can inhibit downstream signaling of Nuclear Factor Kappa B (NF-κB) and Protein 53 (p53) via ERK, consequently delaying the growth of lung cancer cells [11].

**Table 5:** Molecular mechanisms involved in PAQR3 signaling

**Type**	**Downstream pathways**	**Upstream pathways**	**Ref**
Osteosarcoma	p-ERK	-	[7]
Glioma	PI3K/AKT	-	[9]
Lung cancer	Nf-kB/p53	-	[11]
Lung cancer	PI3K/AKT	-	[12]
Hepatocellular carcinoma	-	miR-543/PAQR3↓	[13]
Cervical cancer	EMT, p-AKT, p-ERK	circ_0043280/miR-203a-3p/PAQR3↑	[15]
CRC	EGF/p-ERK, EGF/β-catenin	-	[17]
DLBCL	LDLR/PI3K/AKT, ferroptosis	-	[18]
ALL	Nrf2/ferroptosis	-	[19]
Breast cancer	-	miR-15b/PAQR3↓	[20]
Breast cancer	-	HER2/PAQR3↓	[21]
Breast cancer	-	5-Aza-CdR/PAQR3↑	[22]
ESCA	P27/p21/CYCLD, p-ERK	5-Aza-CdR/PAQR3↑	[23]
ESCA	EMT, p-AKT	-	[24]
ESCA	EMT, p-ERK	-	[25]
Gastric cancer	TGF-β/Smad/EMT	-	[26]
Gastric cancer	p-AKT, p-ERK, EMT	-	[27]
RCC	VEGF/ERK, HIF-1α/p300	-	[28]
Lung cancer	BECN1/Autophagy, EGFR/Autophagy	ATG7/PAQR3↑	[29]
Prostate cancer	p-AKT, p-ERK, EMT	-	[30]
Gastric cancer	EMT	miR-15b-5p/PAQR3↓	[31]
Gastric cancer	EGF/ERK, AKT, insulin	DDB2/PAQR3↓	[32]
Gastric cancer	Twist1/EMT	-	[33]
Colon cancer	PI3K/AKT	P6-55/PAQR3↑	[36]

Abbreviations: CRC: Colorectal cancer; RCC: Renal cell carcinoma; ESCA: Esophageal cancer; ALL: Acute lymphoblastic leukemia; DLBCL: Diffuse large B-cell lymphoma; EMT: Epithelial-mesenchymal transition.

### The PAQR3/phosphatidylinositol 3-kinase (PI3K)/AKT serine/threonine kinase (AKT) signaling axis

The PI3K/AKT pathway is a well-established signaling cascade that promotes cell survival and insulin secretion [38, 39]. Research has elucidated a significant relationship between *PAQR3* and the PI3K/AKT signaling pathway [9, 12, 15, 18, 24, 27, 30, 36]. For instance, Li et al. found that *PAQR3* overexpression inhibits PI3K/AKT signaling by reducing PI3K phosphorylation (p-PI3K) and AKT phosphorylation (p-AKT) without significantly affecting the overall protein levels of PI3K and AKT, thus inhibiting the growth of lung cancer cells A549 and H1299 [12]. In diffuse large B-cell lymphoma U2932 cells, *PAQR3* may impede cell progression through the Low-Density Lipoprotein Receptor (LDLR)/PI3K/AKT signaling pathway by modulating the expression levels of LDLR, p-AKT, and p-PI3K [19]. Moreover, studies indicate that *PAQR3* expression is associated with insulin signaling mechanisms [32]. *PAQR3* inhibits insulin-stimulated phosphorylation of p-AKT and Glycogen Synthase Kinase 3 Beta (GSK3β), thereby influencing the growth and metastasis of gastric cancer [32].

### The PAQR3/Epithelial-mesenchymal transition (EMT) signaling axis

The EMT process is critically linked to cancer metastasis [40]. *PAQR3* contributes to the metastasis of cervical cancer, esophageal cancer, gastric cancer, and prostate cancer through the EMT process [15, 24, 25, 27, 30, 31, 33]. For instance, Huang et al. reported that *PAQR3* overexpression suppresses vimentin expression during EMT while promoting E-cadherin and Zonula Occludens-1 (ZO-1) expression, thereby inhibiting migration of prostate cancer cells PC3 and DU145 [30]. Bai et al. noted that *PAQR3* overexpression enhances E-cadherin expression while inhibiting N-cadherin expression, consequently reducing migration of esophageal cancer cells [24, 25]. Wu et al. demonstrated that *PAQR3* inhibits the expression levels of snail, vimentin, Transforming Growth Factor Beta 1 (TGF-β1), Phosphorylated SMAD Family Member 2 (p-SMAD2), and Phosphorylated SMAD Family Member 3 (p-SMAD3) within the TGF-β/SMAD/EMT signaling pathway, thereby promoting E-cadherin expression and inhibiting gastric cancer progression [26].

### Other downstream signaling mechanisms

Dysregulation of *PAQR3* may also impact ferroptosis through interactions with nuclear factor erythroid 2-related factor 2 (Nrf2), Epidermal Growth Factor (EGF)/β-catenin signaling, and autophagy, thereby influencing cancer progression [17–19, 26, 28, 29, 36]. For example, Wang et al. reported that *PAQR3* inhibits nuclear accumulation of β-catenin in colorectal cancer SW-480 cells, thus delaying tumorigenic potential [17]. Additionally, it may promote ferroptosis in diffuse large B-cell lymphoma and acute lymphoblastic leukemia [18, 19]. Specifically, Song et al. observed that *PAQR3* overexpression suppresses glutathione (GSH) levels in diffuse large B-cell lymphoma cells while promoting Malondialdehyde (MDA), Reactive Oxygen Species (ROS), and Fe2+ levels [18]. Jin and Tong found that *PAQR3* overexpression enhances MDA, Dichlorofluorescein (DCF), and Fe2+ levels in acute lymphoblastic leukemia cells [19]. Furthermore, *PAQR3* can inhibit the Hypoxia-Inducible Factor 1a (HIF-1α)/E1A Binding Protein p300 (p300), Beclin 1 (BECN1)/autophagy, and Epidermal Growth Factor Receptor (EGFR)/autophagy pathways to suppress progression in lung cancer and renal cell carcinoma [28, 29].

### Decreased PAQR3 expression as a biomarker for poor prognosis in cancer patients

Table 6 presents indicators related to the prognosis and pathological features associated with *PAQR3* overexpression in cancer patients. Notably, overexpression is linked to favorable characteristics concerning metastasis, pathological stage, tumor size, and diagnosis [7, 8, 10, 11, 14, 16–18, 21, 23, 24, 26, 27]. Specifically, decreased *PAQR3* expression levels correlate significantly with shorter overall survival (OS) in patients with lung cancer, hepatocellular carcinoma, diffuse large B-cell lymphoma, breast cancer, esophageal cancer, and gastric cancer [10, 14, 18, 21, 24, 26]. Furthermore, reduced *PAQR3* levels are associated with shorter disease-free survival (DFS) in patients with hepatocellular carcinoma, breast cancer, esophageal cancer, and gastric cancer [14, 21, 24, 27]. Additionally, diminished *PAQR3* expression correlates significantly with pathological staging, subtype, tissue differentiation, metastasis, tumor size, and diagnosis in lung cancer patients [10, 11]. It is also linked to factors such as Helicobacter pylori, venous invasion, invasion depth, lymph node metastasis, pathological stage, age, tumor size, tumor differentiation, and distant metastasis in gastric cancer patients [26, 27]. The relationship between *PAQR3* expression and patient prognosis may be confounded by other factors, including co-existing genetic mutations, the patient’s immune status, and concurrent medication use. Future studies should consider these variables to accurately assess the prognostic value of *PAQR3*.

**Table 6:** Clinicopathological features related to PAQR3 in cancer

**Type**	**PAQR3 expression**	**Prognosis**	**Clinical characteristics**	**Ref**
Osteosarcoma	Down	-	Metastasis↑	[7]
Thyroid cancer	Down	-	Extrathyroidal-extension	[8]
Lung cancer	Down	OS↓	Pathological stages↑, tissue differentiation↓, metastasis↑	[10]
Lung cancer	Down	-	Tumor size↑, diagnosis	[11]
Hepatocellular carcinoma	Down	OS↓, DFS↓	AFP↑, tumor size↑, tumor grade↓, recurrence↑	[14]
CRC	Down	-	Tissue differentiation↓, lymph node metastasis↑, depth of invasion↑	[16]
CRC	Down	-	Gender, tumor grade↓	[17]
DLBCL	Down	OS↓	-	[18]
Breast cancer	Down	OS↓, DFS↓	Tissue differentiation↓, pathological stage↑, Her2 status	[21]
ESCA	Down	-	Pathological stage↑, lymph node metastasis↑	[23]
ESCA	Down	OS↓, DFS↓	Nationality, tumor size↑, lymph node metastasis↑, recurrence↑	[24]
Gastric cancer	Down	OS↓	Helicobacter pylori, venous invasion↑, invasion depth↑, lymph node metastasis↑, pathological stage↑	[26]
Gastric cancer	Down	DFS↓	Age, Helicobacter pylori, tumor size↑, tumor differentiation↓, venous invasion↑, lymph node metastasis↑, invasion depth↑, pathological stage↑, distant metastasis↑	[27]

Abbreviations: CRC: Colorectal cancer; ESCA: Esophageal cancer; DLBCL: Diffuse large B-cell lymphoma; OS: Overall survival; DFS: Disease-free survival; AFP: Alpha-fetoprotein.

## Conclusion

Research has established that *PAQR3* functions as a tumor suppressor gene in cancer, and activation of *PAQR3* expression may enhance patient prognosis. This is linked to various signaling mechanisms, including PI3K/AKT, EMT, ferroptosis, and Ras/Raf/MEK/ERK pathways, and is regulated by miR-543, miR-203a-3p, miR-15b, HER2, and 5-Aza-CdR, thereby influencing cancer cell growth and metastasis (Table 5). The anti-tumor mechanisms of *PAQR3* exhibit significant heterogeneity. In certain cancer types, such as diffuse large B-cell lymphoma, *PAQR3* enhances ferroptosis through the PI3K/AKT pathway [18]. In contrast, in other cancers, such as gastric cancer, it may operate via different pathways. These differences may be attributed to cancer-specific expression patterns, tissue-specific microenvironments, mutation backgrounds, and upstream regulatory factors. Furthermore, while epigenetic drugs like 5-Aza-CdR can demethylate and activate *PAQR3* expression, their clinical application faces challenges, including strong toxicity and the development of drug resistance, underscoring the urgent need for more precise epigenetic intervention strategies.

Despite the progress, studies on *PAQR3* remain in preliminary stages. Future research should focus on the upstream and downstream mechanisms of *PAQR3* to deepen the understanding of its pathogenesis and ultimately impede disease progression. Additionally, anti-cancer treatments targeting *PAQR3* encounter various challenges. First, the heterogeneity of the underlying mechanisms necessitates the design of specific treatment strategies tailored to different cancer types, alongside precise regulation of the *PAQR3* pathway in conjunction with tumor molecular typing and microenvironment characteristics. Second, the development of drugs targeting *PAQR3* must address critical issues, including low delivery efficiency and poor stability. Moreover, the *PAQR3* pathway interacts with multiple signaling networks, raising concerns about potential off-target effects from targeted therapies. Enhancing treatment safety through highly selective delivery systems (e.g., nanocarriers or conditionally activated gene editing tools) is essential. Ultimately, the combined targeting of upstream regulatory factors (e.g., HER2 inhibitors) or downstream effector pathways (e.g., ferroptosis inducers) may represent a crucial approach to improve efficacy and overcome drug resistance. Future research could utilize patient-derived organoids to more accurately replicate the *in vivo* tumor microenvironment and investigate the roles of *PAQR3*. Overall, this review summarizes the mechanisms and clinical significance of *PAQR3*, providing a novel theoretical foundation and direction for cancer treatment.

## Data Availability

All data were obtained from published literature, open-access resources, or the corresponding author.
